# Use of a novel shockwave trode results in better patient acceptance in awake canine patients treated for musculoskeletal disease

**DOI:** 10.3389/fvets.2023.1249592

**Published:** 2023-08-09

**Authors:** Gina L. Joseph, Felix M. Duerr, Tianjian Zhou, Lindsay H. Elam

**Affiliations:** ^1^Department of Clinical Sciences, Colorado State University, Fort Collins, CO, United States; ^2^Department of Statistics, Colorado State University, Fort Collins, CO, United States

**Keywords:** canine, musculoskeletal, extracorporeal, shockwave, orthopedic, awake

## Abstract

**Introduction:**

Extracorporeal shockwave therapy (ESWT) is used as a treatment option for several musculoskeletal pathologies in dogs. When performing ESWT using electrohydraulic devices, sedation is commonly recommended due to the noise and discomfort associated with the treatment. The aim of this study was to compare the tolerance of ESWT delivered by a standard or novel trode in awake canine patients with musculoskeletal disease.

**Materials and methods:**

This was a prospective, blinded clinical trial in which dogs with musculoskeletal disease received awake treatment with ESWT with a gradually increasing energy protocol using both standard and novel trodes with an electrohydraulic generator in a randomized fashion. Noise reactivity and tolerance to treatment as measured in number of shocks and energy level achieved were recorded.

**Results:**

Forty client-owned dogs with pathology affecting the hips, stifles, elbows, or shoulders were enrolled. Thirty-three dogs completed all three treatment sessions, three dogs completed two sessions, and four dogs completed one session. There was evidence of improved patient tolerability with the novel trode, based on an increased average number of shocks delivered (*n* ± SD = 848 ± 334 for novel trode; *n* ± SD = 767 ± 358 for standard trode; *p* = 0.0384) and higher average treatment energy level achieved (E ± SD = 6.5 ± 2.5 for novel trode; E ± SD = 5.3 ± 2.8 for standard trode; *p* = < 0.001). Decreased noise reactivity was found to be positively correlated with tolerability of shockwave treatment (energy level: *p* = 0.0168; number of shocks: *p* = 0.0097).

**Discussion:**

Administration of electrohydraulic ESWT is feasible in select awake patients using a gradually increasing energy protocol, and the tested novel shockwave trode is better tolerated than the standard trode. Further studies are required to determine the efficacy of the novel trode, and if gradually increasing energy protocols are clinically equivalent to current standard protocols that employ a consistent energy level.

## 1. Introduction

Extracorporeal shockwave therapy (ESWT) was developed in the 1970s as a non-invasive method for treating kidney and bladder stones in humans (1). Its use has since extended beyond lithotripsy, and it is now used for a number of orthopedic conditions (2, 3). In canines, ESWT is used to treat elbow, stifle and hip osteoarthritis (4–8), and shoulder tendinopathies (9–12). In addition, ESWT has been shown to accelerate bone healing (13) and improve weight bearing after tibial plateau leveling osteotomy (14).

Shockwave therapy can be delivered as either a focused pressure wave or an unfocused, radial wave (3). Focused shockwaves can be generated via three different types of energy sources: electromagnetic, electrohydraulic, and piezoelectric (15, 16). Each focused shockwave is generated in a handpiece, termed a trode, and delivered into tissues through a coupling medium. When the shockwaves encounter an interface with a change in tissue density, such as between bone, tendon, or ligament, they stimulate mechanotransduction. This has been suggested to lead to increased vascularization, the promotion of collagen production and organization, and tissue regeneration (15, 16). Shockwaves are engineered to reach a targeted tissue depth which varies between generation methods, machines, and trodes. Standard electrohydraulic shockwave machines tend to generate a higher acoustic energy wave that can penetrate more deeply into tissues (3).

Many manufacturers recommend the use of ear plugs and sedation for the patients due to the discomfort and noise produced during treatment (3). To the authors' knowledge, the currently published research involving focused shockwave in canines have all been performed under sedation or general anesthesia, however anecdotally in dogs and horses, treatments have been administered without sedation (3). While generally safe when appropriate protocols are applied, sedation is not without risk to the patient and involves additional time and cost demands of the client and veterinary staff (17–23). While shockwave therapy has been associated with minor adverse side effects, the main risk of severe complications associated with the treatment results from the sedation. These factors have led manufacturers to develop new methods of shockwave generation and delivery to canine patients. One such method involves increasing the focal dimensions over which the energy is delivered (24, 25). A recently released novel trode is proposed to decrease local discomfort at the skin-trode interface while still delivering focused energy to the tissues and increasing the volume of treated tissues. However, to the authors' knowledge, it is currently unknown if this novel trode would allow for treatment without sedation. Therefore, the aim of this study was to compare the tolerance of ESWT delivered by an electrohydraulic generator using a standard and novel shockwave trode in awake canine patients with musculoskeletal disease affecting the hips, stifles, elbows, or shoulders.

## 2. Materials and methods

This was a prospective, blinded, randomized clinical trial, and the study design was approved by the Institutional Animal Care and Use Committee and Clinical Review Board (IACUC: #1744 at Colorado State University) and all institutional regulations and guidelines were followed. Client-owned dogs weighing at least 10 kg with confirmed musculoskeletal disease affecting the hips, stifles, elbows, or shoulders were recruited. Owners were informed of the study requirements and consented to treatment at the time of enrollment. Musculoskeletal disease was confirmed prior to enrollment via review of available diagnostic imaging (radiographs, ultrasound, and/or computed tomography). Participation in the study was only offered if the primary clinician determined that the patient would benefit from extracorporeal shockwave therapy and the owners were interested in pursuing treatment. Patients were excluded if they had septic, immune-mediated or neoplastic disease affecting the joint(s) to be treated. Patients were also excluded if they had behavioral conditions requiring medications, were on any behavior modifying medications, had been sedated within 24 h, or were not amenable to gentle handling/restraint techniques. Specifically, dogs that were overly aggressive or fearful on preliminary exam or would hide and refuse to leave the corner of the exam room were excluded.

**Figure 1:**
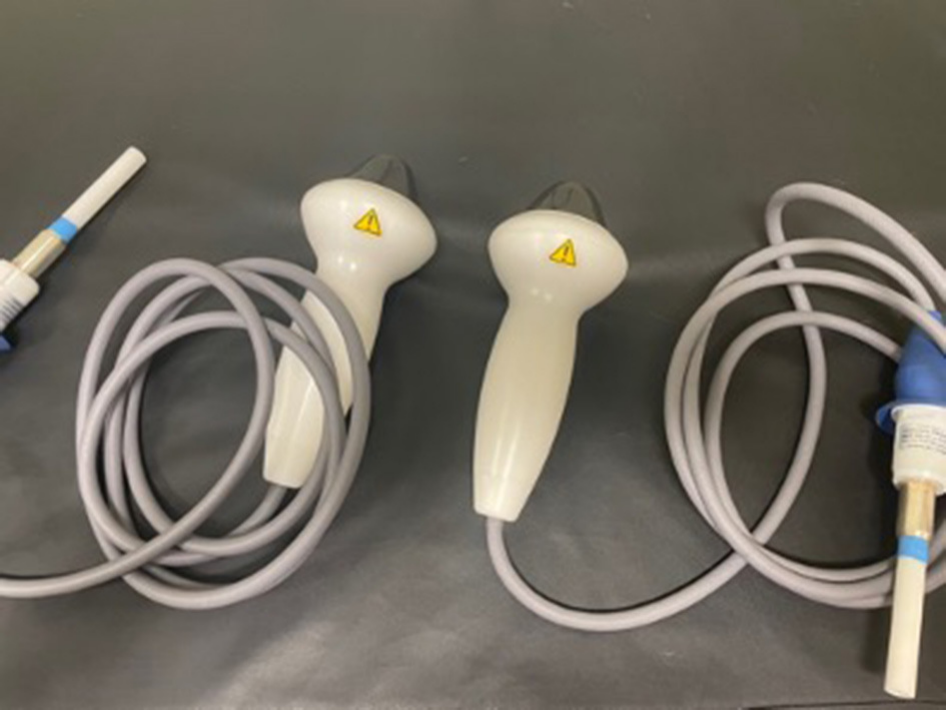
The novel and standard shockwave trodes.

Dogs received treatment with both a standard and novel trode for a total of three treatments. Each treatment was performed ~2–4 weeks apart. Three treatments were chosen because it falls within the current protocol at the authors' institution and allowed for blinding of the single observer (i.e., to avoid knowing the following treatment would be the alternate trode if only two treatments were performed). At the first visit, each patient was randomly assigned to a trode order protocol determined by rolling a standard six-sided die (Bicycle, The United States Playing Card Company, 2018), with each number corresponding to a specific protocol (Table 1). A single clinician (GJ) delivered all treatments and was blinded to which trode was being used. The trodes were grossly identical in emitted sound, appearance, and dimensions and labeled only on the part of the trode that plugged into the main unit (Figure 1). Trodes were plugged into the main unit by a veterinary technician, allowing for blinding of the observer.

**Table 1:** Trode Order Protocol Allocation.

**Number on die**	**Treatment protocol**
1	NNS
2	NSN
3	SNN
4	SSN
5	SNS
6	NSS

S, standard trode; N, novel trode.

At each treatment session, the patient was allowed to be positioned in either lateral recumbency, sitting or standing based on their comfort and where treatment was being applied. Minimal manual restraint was applied so that patients could easily move around in response to the trode. All patients were offered food (peanut butter, kibble, treats) while receiving treatment. Treatments were performed in the same space in the hospital to control for any behavioral response due to changes in the environment. Prior to treatment at each visit, the haircoat was evaluated and clipped if it exceeded 1/4”.

Each patient was initially evaluated for noise reactivity by discharging ~10 shocks both at a far distance (at least five feet away) and near distance (immediately adjacent to the patient). Noise reactivity was scored by the blinded clinician on a numerical 0–3 scale. A score of 0 indicated the patient did not react to the trode. A score of 1 was considered a mild reaction (i.e., the patient would look at the trode but could be easily distracted with food). A score of 2 was given if the patient had a moderate reaction (i.e., the patient would not take or stop taking food, body shaking, tucking tail, but did not try to actively get away). A score of 3 was given if the patient showed a severe reaction (i.e., actively tried to get away from the trode). Noise reactivity scores were recorded at each treatment with the higher score being recorded if near and far scores differed. Noise reactivity was tested and recorded at each session.

Consistent anatomic landmarks were used for treating patients to allow for a standardized treatment area. With each treatment, the center of the trode head was directed perpendicular to the intended tissue to receive treatment. For the shoulder, the protocol used by Leeman et al. was followed (11). For the hip, the treatment area was a circular region with a radius of 3 cm with the center of the greater trochanter marking the center of the treatment area. Both the hip and shoulder were treated from the lateral aspect only to maintain patient comfort during treatment. Treatment for the elbow was performed circumferentially with the treatment area measuring the distance from the lateral humeral epicondyle to olecranon caudally and centering the treatment at the level of the epicondyles. Stifles were treated in a U-shape along cranial, medial and lateral aspects of the joint with the center of the trode head pointing toward the middle of the imaginary cube created in the area between the fabella, patella, head of fibula, and tibial tuberosity.

The shockwave device (Zomedica PulseVet^®^ ProPulse^®^. Ann Arbor, MI; trodes: ProPulse R05 and X-trode. Ann Arbor, MI) was programmed to deliver 1,000 shocks at a rate of 360 shocks/minute. Isopropyl alcohol and a coupling gel (LithoClear^®^ Scanning Gel, Next Medical Products. Branchburg, NJ) was applied to the treatment area prior to initiation of treatment. Ear protection was not required to be worn by the clinicians and staff during treatment but were available for use if desired. Treatment was started and the patient's response was evaluated by the single blinded operator using a modified CMPS based on Reid et al. (26) (Table 2). Initial scoring was performed during the initial 200 shocks at an energy level of E2 with subsequent scoring approximately every 100 shocks after. Based on a patient's score, the energy level was either increased (score of 0), left the same (score of 1), decreased (score of 2), or treatment was stopped (score of 3). If a patient received a score of 2 at any point during treatment, the energy level was immediately decreased and reassessed within the next 100 shocks. If a patient received a score of 3, treatment was immediately discontinued. Treatment energy level was adjusted until a total of 1,000 shocks were delivered, or treatment was discontinued. After treatment, the patients were immediately discharged back to their owners.

**Table 2:** Glasgow Composite Measure Pain Scale.

**Patient behavioral signs**	**Assigned score**	**Treatment response**
• Vocalization: Quiet • Response to trode/touch: No reaction • Posture/Activity: Quiet/comfortable	0	Go up in energy level
• Vocalization: Quiet • Response to trode/touch: Look at trode/around • Posture/Activity: Unsettled/restless	1	Stay at same energy level
• Vocalization: Crying/ whimpering, groaning • Response to trode/touch: Flinch, growl/guard • Posture/Activity: Hunched/tense	2	Go down in energy level
• Vocalization: Screaming • Response to trode/touch: Snap, cry • Posture/Activity: Rigid/Attempting to leave	3	Stop treatment

A power calculation based on the tolerability of treatment energy was performed on preliminary data from 10 patients using Excel (Microsoft Corporation, Microsoft Excel. 2018. Redmond, WA). With a minimum detectable difference of two levels of energy and a significance level of 0.05, a desired sample size of 40 resulted in a power of 0.9919. The outcome measures were analyzed using a linear mixed model that was fit separately for each response variable (energy and number of shocks). Each individual dog was considered a random effect to account for the correlation among multiple repeated measures of the same subject. The type of trode, noise reactivity and joint treated were included in the model as fixed effects. The statistical analyses were performed using the R statistical software (R Core Team, R: lme4. 2022. Vienna, Austria). Residual diagnostic plots were used to evaluate model assumptions, and no obvious violations of modeling assumptions were identified. A *p* < 0.05 was used as a threshold for declaring statistical significance. Furthermore, the Holm procedure for multiple testing was considered for the two primary comparisons of energy and number of shocks (27, 28) to control the family-wise error rate at 0.05. The Holm procedure was implemented by first comparing the *p*-value for energy level (*p* = 0.0007) to 0.05/2, and then comparing the *p*-value for number of shocks (*p* = 0.0384) to 0.05. The comparisons were still significant under the Holm procedure and did not affect the conclusions. For all other comparisons and associations, no multiplicity adjustments were performed, and the *p*-values should be interpreted for descriptive purposes (29).

## 3. Results

Forty client-owned dogs with diagnosed pathology affecting the treated joints and/or associated soft tissues (stifles: *n* = 9, hips: *n* = 9, shoulders: *n* = 11, and elbows: *n* = 11) were enrolled in the study. None of the enrolled patients had undergone surgery of the affected joint within the 6 months prior to enrollment or had received any local anesthetic at the treatment site. The average age of the enrolled patient was 9.2 years (SD ± 3.95; range: 2–14.8) with most patients being sterilized (spayed: *n* = 19, neutered: *n* = 17, intact female: *n* = 2, intact male: *n* = 2). The most common breeds were Labrador Retrievers (*n* = 11), and mixed breed dogs (*n* = 9), followed by, American Pit Bull Terriers (*n* = 2), Border Collies (*n* = 2), German Shepherd Dogs (*n* = 2), and Golden Retrievers (*n* = 2). The remaining breeds included Greater Swiss Mountain Dog (*n* = 1), Rottweiler (*n* = 1), English Bulldog (*n* = 1), Australian Shepherd (*n* = 1), Bernese Mountain Dog (*n* = 1), Cane Corso (*n* = 1), French Bulldog (*n* = 1), Pembroke Welsh Corgi (*n* = 1), Mastiff (*n* = 1), Labradoodle (*n* = 1), and Vizsla (*n* = 1). Average patient weight was 29.7 kg (SD ± 8.08; range: 11–51.2 kg).

Thirty-three dogs completed all three treatment sessions, three dogs completed two sessions, and four dogs completed one session. Of the patients who did not complete the full treatment schedule, two were treated with both trodes, three with the novel trode alone and two with the standard trode alone. Reasons for participants to not complete the three scheduled treatment sessions included a lack of perceived patient improvement by the owners (*n* = 5), lost to follow up (*n* = 1), or euthanasia unrelated to the study (*n* = 1).

In total, treatment was initiated 53 times with the novel trode (average energy ± SD = 6.5 ± 2.5; average number of shocks administered ± SD = 848 ± 334) and 56 times with the standard trode (average energy ± SD = 5.3 ± 2.8; average number of shocks administered ± SD = 767 ± 358). When evaluating for tolerated energy level, there was evidence of improved tolerability with the novel trode after adjusting for noise reactivity and the joint treated (*p* = 0.0007). When further evaluating for noise reactivity and energy tolerability, there was evidence of correlation between the two (*p* = 0.0168): per unit of increased noise reactivity there was an estimated 0.69 unit (standard error: 0.284) lower tolerability of shockwave energy.

Similarly, when comparing number of shocks tolerated between the two trodes, there was evidence of improved tolerability with the novel trode after adjusting for noise reactivity and joint treated (*p* = 0.0384). There was also a positive correlation between noise reactivity and number of shocks, after adjusting for joint treated and treatment group (*p* = 0.0097): per unit of increased noise reactivity, patients tolerated an estimated 96.077 (standard error: 36.442) fewer shocks.

When comparing joints treated (comparison of all four groups together) using a one-way analysis of covariance (ANCOVA), there was no statistically significant difference in patients' tolerability of energy or number of shocks, after adjusting for treatment group and noise reactivity (energy: *p* = 0.1012; number of shocks: *p* = 0.1641). Pairwise comparison between the four joints (Tables 3, 4), however, suggested that hips had the highest tolerability and shoulders had the lowest tolerability. Since the pairwise comparisons were performed after a non-significant ANCOVA, the *p-*values associated with the contrasts presented in Tables 3, 4 should only be interpreted for descriptive purposes.

**Table 3:** Pairwise joint comparison for shockwave energy.

**Contrast**	**Estimate**	**SE**	***p*-value**
Hip—Elbow	0.9	0.9	0.340
Shoulder—Elbow	−1.4	0.9	0.137
Shoulder—Hip	−2.2	0.9	0.015
Stifle—Elbow	−0.5	0.9	0.623
Stifle—Hip	−1.3	0.9	0.154
Stifle—Shoulder	0.9	0.9	0.320

**Table 4:** Pairwise joint comparison for number of shocks.

**Contrast**	**Estimate**	**SE**	***p*-value**
Hip—Elbow	143.1	122.1	0.249
Shoulder—Elbow	−123.1	121.8	0.318
Shoulder—Hip	−266.2	119.2	0.032
Stifle—Elbow	69.6	126.5	0.586
Stifle—Hip	−73.5	124.4	0.558
Stifle—Shoulder	192.7	122.0	0.123

The only minor adverse event reported by a single owner was skin irritation after the initial treatment. The visit included clipping of the haircoat and resolved without treatment per the owner. No long-term adverse effects were reported by the clients or noted on veterinary examinations during the course of the study.

## 4. Discussion

Based on the results of the present study, awake shockwave therapy with a gradually increasing energy protocol is feasible in a selected group of patients with musculoskeletal disease affecting the shoulders, elbows, hips, and stifles. Furthermore, treatment using the novel trode is better tolerated both in terms of energy level reached and number of shocks delivered compared with the standard trode. Dogs in our study population, on average, were able to tolerate ~1 level of higher energy and ~80 additional shocks from the novel trode compared to the standard trode. Our study also showed a relationship between noise reactivity and awake shockwave tolerance, something that has not previously been reported. Further research is needed to evaluate if a gradually increasing energy treatment protocol is equally effective to a consistent energy level protocol. Additionally, further research is needed to evaluate the efficacy of the novel trode in comparison with a standard trode.

Although the data did not show a definitively higher tolerability for a single joint compared with the other three tested, it did suggest that the hips were the most highly tolerated joint. Possible explanations for this could be that the soft tissues surrounding the hips, namely muscle bellies, helped provide “padding” to the affected joint and surrounding major nerves. The remaining three joints (shoulders, elbows, knees) have less soft tissue coverage comparatively, which may result in more discomfort during treatment. Pairwise comparison also suggested lower tolerability for patients treated for shoulder pathologies. It is possible this was observed because the shoulders are physically closer to the patient's head and ears and the noise or physical presence of the trode near the head could have led to decreased tolerability. In addition, there was a higher prevalence of soft tissue pathology in the shoulder patient population, so it is possible that treatment of soft tissue pathologies with shockwave is more painful than degenerative joint disease. In a recent survey of members of the American Association of Equine Practitioners, the majority of respondents that used shockwave reported that “equine patients were moderately to completely tolerable of ESWT, regardless of the body region” (30). In human studies, the patients' tolerance can be a factor in the energy and/or number of shocks delivered during a treatment (1, 31). To the authors' knowledge, there have not been human studies evaluating tolerance of shockwave at different anatomic locations. Adverse effects reported in people for treatment of musculoskeletal disease include local pain, erythema, bruising, hematoma formation, nerve irritation, superficial edema, and even systemic signs including headaches and migraines (2, 32–35).

Shockwave therapy has been associated with minor adverse side effects in canines including pain during treatment, local bruising, ecchymosis and/or petechiation, hematoma formation and local swelling (3, 4, 36). Patients who were assigned higher modified Glasgow Composite Measure Pain Scale (CMPS) scores can be presumed to have had greater pain at the treatment area, consistent with reports in previous studies (4). The only other side effect noted during the study was skin irritation after the first treatment, but this was suspected to be related to coat clipping rather than the shockwave therapy itself as the irritation did not recur with subsequent treatments. Given the low incidence of adverse events, a comparison between the two trodes was not feasible.

The results of this study help support the use of shockwave for treating awake patients. While patients with musculoskeletal disease may be sedated for diagnostics or other routine outpatient procedures, it is not without its risks. Commonly used sedation medications include opioids and alpha-2 agonists, both of which have been shown to have negative cardiopulmonary effects on even healthy dogs (17–21). A survey of owners in the United States and Canada revealed that over 20% of owners strongly disagreed with the use of sedation during even routine exam (22).

The novel trode technology is described by the manufacturer as including a proprietary change to the reflector geometry designed to reduce the peak focal energy and spread the 5 MPa focal zone, which is considered the threshold for therapeutic effect of shock wave on tissue (24, 25, 37). This change is intended to reduce the pain of treatment while impacting a greater volume of tissue with the treatment. This approach, however, still differs from radial shockwave, which generates pressure waves that extend outwards equally from the generation source and lose energy at a rate proportional to radius^−1^. This leads to lower amplitude waves that have a lower velocity, by two orders of magnitude, than the speed of sound in tissue (16).

There are several limitations to the present study, including the small sample size, particularly given that treatment was not limited to one specific joint. Another limitation was the subjective nature of the outcome measures. The authors attempted to decrease subjectivity through the use of a modified CMPS that has been validated in dogs for acute pain (38). While this judgment is subjective, to the authors' knowledge, there is no validated, objective method to determine acute pain that could be used in the study setting. To minimize bias and confounding factors, a single, blinded clinician performed all the scoring.

Because our study was focused on tolerability of treatment rather than clinical response, clinical metrology instruments or objective gait analysis were not used as outcome measures. Additional physiologic measures of stress or pain such as continuous blood pressure or heart rate monitoring were not used due to the potential to affect behavior response during collection and recording. Another potential limitation is that the patients' medical management protocols were not standardized due to the variety of conditions treated as well as severity. It is possible that patients who were receiving anti-inflammatory and/or analgesic medications may have shown higher tolerance to shockwave compared with patients not on these medications. Additionally, the severity of a patient's overall pain may have also affected their tolerance to therapy or noise sensitivity (39).

The inclusion criteria resulted in patients who were amenable to gentle restraint without the need for sedative or anxiolytic medications, which likely created bias toward patients with better tolerance. This also likely explains the high tolerability, even with the standard trode. The described exclusion criteria were chosen to help minimize additional confounding factors such as reactivity to restraint itself instead of the treatment or variations in serum plasma levels of medications due to differences in time of administration or dosing. As such, the results from this study cannot be extrapolated to more stressed patients. From a clinical perspective however, a patient who is not tolerating initial awake treatment could be given pre-visit oral anxiolytic medication at the next visit or the clinician could use earplugs in patients who appear noise reactive. Occupational Safety and Health Administration (OSHA) guidelines require ear protection for employees who are exposed to average noise levels above 85 decibels over an 8 h period (40). Per manufacturer recommendations, ear protection is recommended if sustained treatment is being performed. As our blinded clinician and staff were only exposed to shockwave treatment lasting no more than 4 min at a time and only a maximum of four treatments per day, ear protection was not required but available if desired. These recommendations should be considered by clinicians if longer treatments or multiple daily treatments are performed.

It was outside the scope of the study to compare the clinical efficacy of the novel trode to the standard trode when used with an electrohydraulic generator. Future blinded, prospective studies using objective outcomes are needed to determine if the novel trode provides different clinical outcomes compared with the standard trode. The gradually increasing energy protocol has also never been evaluated for clinical efficacy and additional prospective studies could be performed to evaluate whether its efficacy compared to a static, high energy treatment protocol.

The presented results support the use of ESWT in awake patients. The modified scale used in this study was originally developed to assess acute pain in patients, which is one of the primary limiting factors for treatment. The results show that while the novel trode was overall better tolerated than the standard trode, the standard trode still had better than anticipated tolerability, so either trode could be used to treat awake patients based on the clinician's discretion. It cannot be overstated that the purpose of this study was purely focused on tolerability of awake shockwave and should not be used to make conclusions regarding clinical efficacy of either trode or the protocol used.

## Data availability statement

The original contributions presented in the study are included in the article/Supplementary material, further inquiries can be directed to the corresponding author.

## Ethics statement

The animal studies were approved by Institutional Animal Care and Use Committee and Clinical Review Board (IACUC: #1744 at Colorado State University). The studies were conducted in accordance with the local legislation and institutional requirements. Written informed consent was obtained from the owners for the participation of their animals in this study. No potentially identifiable images or data are presented in this study.

## Author contributions

GJ contributed to study design, data collection, and primary manuscript drafting. FD and LE contributed to study design and manuscript editing. TZ contributed to data analysis and manuscript editing. All authors contributed to the article and approved the submitted version.
