# Supplementary material for: Use of a novel shockwave trode results in better patient acceptance in awake canine patients treated for musculoskeletal disease
Source: Front Vet Sci. 2023 Aug 9;10:1249592. doi: 10.3389/fvets.2023.1249592 (PMC10445390; doi:10.3389/fvets.2023.1249592)
Supplement: Supplementary file 1 [file Data_Sheet_1.PDF]

Supplementary Material

Use of a novel shockwave trode results in better patient acceptance in awake canine patients treated for musculoskeletal disease

Joseph G. L.[1], Duerr F. M.[1], Zhou T.[2], Elam L. H.[1*]

***Correspondence:** Lindsay Elam, Lindsay.elam@colostate.edu

					Treatment session 1				Treatment session 2				Treatment session 3				
PT #	Signalment	Weight (kg)	Joint (Diagnosis)	Maintenance Anti-Inflammatories and/or Analgesics	Trode	Noise Score	E level	Shocks	Trode	Noise Score	E level	Shocks	Trode	Noise score	E level	Shocks	Preferred Trode
1	3.8y FS Greater Swiss Mountain Dog	46	Elbow (Moderate Flexor Enthesopathy)	None	1	0	7	1000	1	0	8	1000	2	0	8	1000	NEUTRAL
2	11.1y FS Border Collie	26.8	Shoulder (Mild Biceps Tendinopathy)	Galliprant	2	0	8	1000	2	0	8	1000	1	0	4	1000	STANDARD
3	10.8 MN MBD	28.1	Hip (Severe Osteoarthritis)	Carprofen, Pregabalin, Methocarbamol	1	0	8	1000	2	0	3	1000	-	-	-	-	NOVEL
4	10.2y FS MBD	16.5	Shoulder (Mild Biceps Tendinopathy)	Carprofen, Gabapentin, Methocarbamol	1	0	8	1000	2	1	2	169	1	3	2	262	NOVEL
5	8.1y FI Rottweiler	38.2	Hip (Severe Osteoarthritis)	Carprofen, Gabapentin, Amantadine	2	0	8	1000	1	0	8	1000	2	0	8	1000	NEUTRAL
6	10.3y MN MBD	21.4	Elbow (Moderate Osteoarthritis)	Meloxicam	2	0	7	1000	2	0	8	1000	1	1	8	1000	NEUTRAL
7	3.3y FS GSD	30.7	Elbow (Moderate Osteoarthritis)	Carprofen	2	1	4	783	1	3	2	0	-	-	-	-	STANDARD
8	9.9y FS English Bulldog	22.8	Elbow (Moderate Osteoarthritis; Moderate Flexor & Extensor Enthesopathy)	Carprofen	1	0	8	1000	2	0	8	1000	2	0	8	1000	NEUTRAL
9	12.9y FS Labrador Retriever	29.9	Shoulder (Moderate Biceps Tendinopathy)	Carprofen	2	1	8	1000	1	1	8	1000	1	1	8	1000	NEUTRAL
10	12.7y FS Australian Sheperd	20.5	Elbow (Mild Osteoarthritis)	Gabapentin	1	0	8	1000	1	0	8	1000	2	0	3	1000	NOVEL
11	6.2y FS Bernese Mountain Dog	39.2	Shoulder (Mild Supraspinatus Tendinopathy)	Carprofen, Gabapentin	2	1	2	123	-	-	-	-	-	-	-	-	-
12	11.4y MN Labrador Retriever	31.1	Shoulder (Moderate Osteoarthritis; Moderate Biceps Tendinopathy)	None	2	1	2	300	1	0	2	852	1	1	2	201	NOVEL
13	11.9y MN Cane Corso	36.2	Shoulder (Minimal Osteoarthritis; Moderate Biceps Tendinopathy)	Galliprant, Amantadine	1	1	8	1000	1	1	8	1000	2	1	8	1000	NEUTRAL
14	10.9y FS Labrador Retriever	28.2kg	Shoulder (Mild Osteoarthritis; Mild Biceps Tendinopathy)	None	1	0	2	262	2	1	2	233	1	1	2	69	NEUTRAL
15	4.1y MN American Pit Bull Terrier	24.6	Stifle (Mild Osteoarthritis)	Carprofen, Gabapentin	1	0	8	1000	-	-	-	-	-	-	-	-	-
16	12.1y FS French Bulldog	11	Elbow (Moderate Osteoarthritis)	Carprofen, Gabapentin	2	0	2	122	-	-	-	-	-	-	-	-	-
17	2.9y FS Labrador Retriever	26.4	Hip (Moderate Osteoarthritis)	None	2	0	7	1000	1	1	8	1000	2	1	8	1000	NEUTRAL
18	14.5y FS Labrador Retriever	31	Hip (Marked Osteoarthritis)	Carprofen, Gabapentin	2	3	3	1000	1	0	8	1000	2	0	8	1000	NEUTRAL
19	13.7y MN MBD	28kg	Stifle (Moderate Osteoarthritis; Mild Lateral Collateral Ligament Enthesopathy)	Prednisone, Gabapentin, Amantidine	2	0	2	99	1	1	7	1000	1	1	8	1000	NOVEL
20	12.2y MN Labrador Retriever	33	Hip (Moderate Osteoarthritis)	Galliprant, Pregabalin	1	0	8	1000	1	0	8	1000	-	-	-	-	-
21	13.6y FS MBD	18	Elbow (Moderate Osteoarthritis)	Galliprant, Gabapentin	1	1	8	1000	1	0	8	1000	2	0	8	1000	NEUTRAL
22	13.7y MN MBD	35.3	Hip (Severe Osteoarthritis)	Carprofen, Gabapentin	2	0	8	1000	1	0	8	1000	2	0	8	1000	NEUTRAL
23	2y FS Golden Retriever	32.4	Hip (Severe Osteoarthritis)	Carprofen	1	0	8	1000	1	0	8	1000	2	0	8	1000	NEUTRAL

24	13.4y FS GSD	33.4	Shoulder (Moderate Supraspinatus Tendinopathies)	Carprofen, Gabapentin	2	0	3	1000	2	0	5	1000	1	0	2	1000	STANDARD
25	14.4y MN MBD	29.3	Elbow (Marked Osteoarthritis; Flexor Enthesopathy)	Carprofen, Gabapentin	2	0	2	245	-	-	-	-	-	-	-	-	-
26	10y MI Labrador Retriever	36.5	Elbow (Moderate Osteoarthritis)	Carprofen, Gabapentin	1	1	8	1000	2	0	8	1000	2	0	8	1000	NEUTRAL
27	8.2y MI Labrador Retriever	27.1	Shoulder (Mild Osteoarthritis)	None	2	0	2	384	2	1	3	590	1	1	2	40	STANDARD
28	5.4y FI Pembroke Welsh Corgi	16.7	Hip (Minimal Osteoarthritis)	None	1	1	8	1000	2	1	5	1000	2	1	8	1000	NEUTRAL
29	12.6y FS Belgian Malinois	29.6	Stifle (Mild Osteoarthritis)	None	1	1	2	203	1	1	7	1000	2	1	2	707	NOVEL
30	6.7y FS Labrador Retriever	27.6	Hip (Moderate Osteoarthritis)	None	2	0	8	1000	1	1	8	1000	2	1	8	1000	NEUTRAL
31	8.7y FS Labrador Retriever	33.3	Stifle (Moderate Osteoarthritis)	None	1	0	8	1000	2	0	5	1000	2	1	7	1000	NOVEL
32	4.9y MN Mastiff	43.2	Stifle (Mild Osteoarthritis)	None	2	1	2	1000	1	2	2	1000	1	2	7	1000	NEUTRAL
33	2.3y MN American Pit Bull Terrier	33.6	Shoulder (Mild Biceps Tendinopathy)	None	2	3	8	1000	2	2	8	1000	1	2	8	1000	NEUTRAL
34	11.4y MN Labradoodle	23.2	Stifle (Moderate Osteoarthritis)	Previcox	2	0	8	1000	2	0	2	200	1	0	8	1000	NEUTRAL
35	13.5y MN Vizsla	30.3	Stifle (Mild Osteoarthritis)	Galliprant, Gabapentin, Amantadine	1	1	8	1000	2	0	8	1000	1	0	8	1000	NEUTRAL
36	4.5y MN MBD	51.2	Elbow (Moderate Flexor Enthesopathy)	Carprofen	2	1	2	260	1	1	8	1000	1	1	8	1000	NOVEL
37	7.6y MN MBD	31.7	Stifle (Moderate Patellar Tendinopathy)	Carprofen	2	3	2	700	2	1	2	347	1	2	8	1000	NOVEL
38	5.1y MN Border Collie	27.4	Shoulder (Marked Biceps Tendinopathy)	Carprofen	1	3	2	51	2	3	2	12	2	2	2	44	NEUTRAL
39	14.8y FS Golden Retriever	25.9	Stifle (Moderate Osteoarthritis)	Carprofen, Gabapentin	2	0	2	607	1	1	8	1000	2	1	8	1000	NOVEL
40	4.4y MN Labrador Retriever	31.1	Hip (Marked Osteoarthritis)	None	2	3	2	11	1	1	2	27	1	1	8	1000	NOVEL

Supplementary Table 1. Recorded noise score, energy level and number of shocks for each treatment session for enrolled patients. Patients listed in red did not complete all study visits. Novel trode – 1, standard trode - 2 . FI - Female Intact. FS - Female Spayed. MI - Male Intact. MN - Male Neutered. MBD - Mixed Breed Dog. GSD - German Shepherd Dog
